# The Conditions of Nux Vomica in Whitlow

**Published:** 1894-01

**Authors:** E. W. Berridge

**Affiliations:** 48 Sussex Gardens, London, W., England


### THE CONDITIONS OF NUX VOMICA IN WHITLOW.

EDITOR OF THE HOMŒOPATHIC PHYSICIAN: In the January number of your journal at page 12, line 24 from the top, Dr. W. L. Reed suggests that the conditions of the whitlow, quoted in *The Guiding Symptoms* under Nux-vomica, have been misprinted.

Permit me to say that the conditions as stated in *The Guiding Symptoms* are quite correct. The case is one cured by myself. It was reported in *The Transactions of The International Hahnemannian Association* for 1892, page 245.

E. W. BERRIDGE, M. D.

48 SUSSEX GARDENS, LONDON, W., ENGLAND.

